# Functional Fitness and Self-Reported Quality of Life of Older Women Diagnosed with Knee Osteoarthrosis: A Cross-Sectional Case Control Study

**DOI:** 10.1155/2015/841985

**Published:** 2015-08-05

**Authors:** Paula Andréa Malveira Cavalcante, Márcio Roberto Doro, Frank Shiguemitsu Suzuki, Roberta Luksevicius Rica, Andrey Jorge Serra, Francisco Luciano Pontes Junior, Alexandre Lopes Evangelista, Aylton José Figueira Junior, Julien Steven Baker, Danilo Sales Bocalini

**Affiliations:** ^1^Postgraduate Program in Physical Education and Aging Science of São Judas Tadeu University (USJT), São Paulo, SP, Brazil; ^2^Department of Physical Education, Nove de Julho University (UNINOVE), São Paulo, SP, Brazil; ^3^Postgraduate Program in Biophotonics Applied to Health Sciences, Nove de Julho University (UNINOVE), São Paulo, SP, Brazil; ^4^Department of Gerontology of Arts, Science and Humanities School of University of São Paulo, São Paulo, SP, Brazil; ^5^School of Science and Sport, University of the West of Scotland, Hamilton, Lanarkshire, UK

## Abstract

*Aim.* Utilizing a cross-sectional case control design, the aim of this study was to evaluate the functional fitness and self-reported quality of life differences in older people diagnosed with knee osteoarthrosis (O) who participated in health promotion groups. *Methods.* Ninety older women were distributed into two groups: control without O of the knee (C, *n* = 40) and a group diagnosed with primary and secondary knee O with grade II or higher, with definite osteophytes (OA, *n* = 50). Functional fitness was evaluated by specific tests, and the time spent in physical activity and quality of life was evaluated by the IPAQ and WHOQOL (distributed in four domains: physical: P, psychological: PS, social: S, and environmental: E) domain questionnaires. *Results.* No differences were found between ages of groups (C: 66 ± 7; OA: 67 ± 9; years). The values of the chair stand test (rep) in the OA (13 ± 5) group were different when compared to C group (22 ± 5). For the 6-minute walk test (meters), the values obtained for the C (635 ± 142) were higher (*P* < 0.01) than the OA (297 ± 143) group. The time spent in physical activity (min) was greater (*P* < 0.001) in the control (220 ± 12) group compared to OA (100 ± 10) group. Higher values (*P* < 0.001) in all domains were found in the C (P: 69 ± 16, PS: 72 ± 17, S: 67 ± 15, E: 70 ± 15) group compared to OA (P: 48 ± 7, PS: 43 ± 8, S: 53 ± 13, E: 47 ± 14) group. *Conclusion.* Our data suggests that knee O, in older women, can promote a decline in time spent performing physical activity and functional fitness with decline in quality of life with an increase in sitting time.

## 1. Introduction

Osteoarthritis (OA) is a chronic, multifactorial disease that leads to progressive functional disability [1] and has been considered one of the most frequent causes of incapacity for work in Brazil [1, 2] and worldwide [3]. Among the joints, knee, hip, hand, foot, and spine [3] are the most common areas for the development of osteoarthritis; however, the knee joint has been the most studied [4].

In histological studies, OA is characterized by focal areas of loss of cartilage in synovial joints by joining capsule thickening and bone hypertrophy with consequent formation of osteophytes and bone sclerosis [3, 5]. Joint pain, tenderness, limitation of movement, crepitus, occasional effusion, and variable degrees of local inflammation are usual signs in the clinical condition of impairment [3, 4]. Around the risk factors, age, female gender, previous joint injury, genetics, and muscle weakness [5] are important factors to pathogenesis; however, many individuals are affected by lifestyle factors such as obesity and lack of physical activity [3, 5].

In the USA it is estimated that 25% of people aged over 65 years suffer from pain and other disabilities associated with OA [6]. In Brazil, there are no precise data on prevalence; however, Coimbra et al. [1] report that it is the most common rheumatic disease found in women in Brazil among individuals over 65 years [6]. As a result, OA will be an important public health problem in future years in Brazil due to the increase in age of the population [7].

The conservative treatment of knee OA requires analgesic therapy for long periods [8]; however, the pharmacological treatment should always be multifactorial [1]. Physical exercise had been used in conjunction with pharmacological treatments. No pharmacologic strategy alone has been identified as an effective method of therapeutic intervention demonstrating pain relief and increased mobility related to patients with knee OA [1, 4, 5, 9, 10]. Additionally, local physical therapy, rehabilitation, and reduction of mechanical stress on joints may provide improvements in pain symptoms and maintain joint function, which mainly reflects the improvement in the quality of life of people affected by the disease [4].

In older patients, functional fitness is impaired by pain, joint stiffness, and muscle atrophy and bone crepitus [4, 5]. In this context, functional fitness evaluation had been highlighted as a relevant strategy utilized for older people [11] and is an indicative measure that individuals have to decide about their functional capacity and that relates to their day to day living and capacity to perform [6, 12, 13]. Another important disclosure has been to address the quality of life [14]. Additionally, studies [6, 13] have shown significant reductions in self-reported quality of life, including pain increase and physical and psychological stress [13]. Considering these points and to address some of the gaps observed in both the literature and experimental studies, the aim of this study was to evaluate the functional fitness and self-reported quality of life differences in older people. Two groups were evaluated, subjects diagnosed with knee osteoarthrosis and a control group without osteoarthrosis of the knee.

## 2. Material and Methods

After approval by the Research Ethics Committee of Nove de Julho University (466/2012), ninety older women (over 60 years) participants of health promotion groups (method of collective and interdisciplinary health intervention, consisting of a group process) were recruited from the Regional Community Adult Day Care facilities by Nove de Julho University and distributed into two groups: without osteoarthrosis of knee (control, *n*: 40) and with primary and secondary knee osteoarthrosis (OA, *n*: 50). All participants had medical examinations and completed questionnaires regarding medical history. All protocols used in the study were performed in accordance with the ethical standards of the Helsinki Declaration.

The following exclusion criteria were assigned: chronic knee pain, knee surgery, current or previous participation in regular exercise programs in the past six months, recent hospitalization, cardiorespiratory disease, severe hypertension, metabolic syndrome and liver or kidney disease, cognitive impairment or progressive conditions with debilitating inability to exercise, recent bone fractures, any knee surgery earlier, and any other medical contraindications for training.

The OA diagnosis was based on clinical and radiographic parameters, in line with the American College of Rheumatology [15] and individual medical history. The radiographic parameters for knee OA diagnosis were established according to the classification of Kellgren and Lawrence [16], with involvement of the knee on or above grade II, conform described on Table 1.

### 2.1. Physical Activity Level

Utilizing the interview strategy, the short form International Physical Activity Questionnaire (IPAQ) was used to estimate physical activity levels. The questions were asked of all subjects on the week preceding the physical activity measures. The questions asked explored the frequency and duration of physical activity including walking, moderate and vigorous physical exercise, and sitting time. Individuals were considered active if they participated in physical activity for more than 150 minutes per week and inactive if their participation levels were less than 150 minutes per week.

### 2.2. Anthropometric Parameters

The anthropometric measures used in this study were similar to those previously reported by our group [14, 17]. Stature was measured to the nearest 0.1 cm using a Cardiomed WCS stadiometer (Curitiba, Brazil). Body mass was measured to the nearest 0.1 kg using a Filizola Personal Line 150 scale (São Paulo, Brazil). Body mass index (BMI) was calculated as follows: BMI = weight/height^2^.

### 2.3. Functional Fitness

Functional fitness evaluation comprised six tests previously reported in the literature to assess physical performance parameters concerning mobility and balance in older adults [12, 14, 17, 18]. The following tests were utilized.

#### 2.3.1. Arm Curl Test

It was used to evaluate upper limb fitness, with the analyzed score as the total number of hand weight curls through the full range of motion.

#### 2.3.2. Chair Stand Test

It was used to evaluate lower limb strength, scored by the number of full stand-ups executed correctly within 30 seconds.

#### 2.3.3. Agility

It was evaluated by the 8-foot up-and-go test (TUG), and the score recorded was considered the shortest time to rise from a seated position, walk eight feet, turn back, and return to the seated position.

#### 2.3.4. Sit and Reach Test

It was used to evaluate the lower body flexibility scored by the shortest distance achieved between the extended fingers and the toe when seated with extended leg and heel resting on the floor.

#### 2.3.5. Static Balance

It was assessed by having subjects stand on one leg for a maximum of 30 seconds on each side. The score was measured, allowing only minimal fluctuations of ankle position or obvious toe clawing, without hopping or upper limb movement. The test was stopped after 30 seconds if hopping occurred, the ankle movement was excessive, or the hanged foot touched the floor or contacted the stance leg/foot.

#### 2.3.6. Functional Exercise Capacity

It was measured by 6-minute walk test [6].

### 2.4. Quality of Life

Quality of life was evaluated by a shortened WHO quality of life questionnaire as outlined previously [14, 17, 19]. The questionnaire comprised 25 questions about several aspects of quality of life, including the following.

#### 2.4.1. Physical Domain

It concerns pain or discomfort, energy or fatigue, sleep, rest, mobility, daily activities, medicine dependency, and job performance.

#### 2.4.2. Psychological Domain

It includes feelings, learning, memory and attention, self-esteem, aspect, spirituality, religiousness, and positive or negative thinking.

#### 2.4.3. Social Domain

It deals with personal relationships, social support, and sexuality.

#### 2.4.4. Environmental Domain

It includes physical security, home environment, financial security, opportunity for information assessment, social or cultural event participation, and activities undertaken during spare time. Each domain was scored from 0 to 100 points, and higher scores represented improvement.

### 2.5. Statistical Analyses

All statistical analyses were performed using SPSS software (v 12.0; IBM, Armonk, NY, USA). The D'Agostino-Pearson test was applied to Gaussian distribution analysis. Analysis of comparisons between groups over time was performed by Student's *t*-test or Mann-Whitney test. Statistical significance was established at *P* < 0.05. Data are expressed as mean ± SEM.

## 3. Results

During functional fitness evaluation phase program, 10 women of OA group dropped out of study due to knee pain during execution of the test. Therefore, fifty women were included on OG group.

The anthropometric parameters are presented in Table 2. The body mass and stature did not differ between groups. However, the BMI of OA group was higher than control group.

Significant differences (*P* < 0.01) were found between time spent in physical activity (control: 220 ± 12 versus OA: 100 ± 10; minutes) and sitting time (control: 1673 ± 532 versus OA: 2675 ± 680; minutes) between groups.

**Figure 1:**
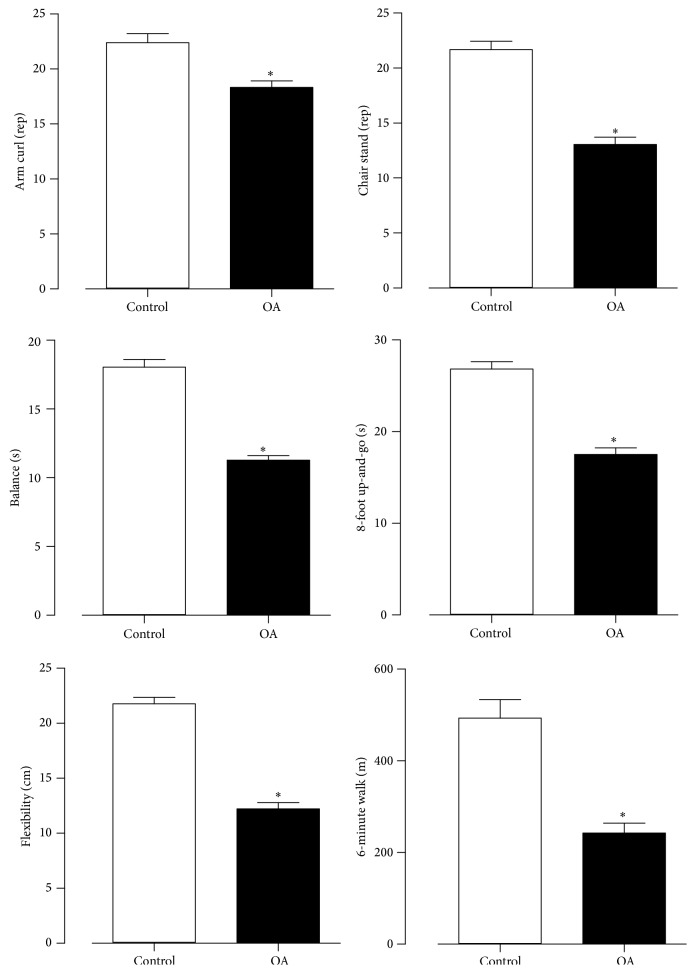
Values expressed in ± SEM of functional fitness test of control and osteoarthrosis (OA) groups. ^*^ *P* < 0.05.

Functional fitness scores are outlined in Figure 1. Statistical differences (*P* < 0.01) were found between groups between the chair stand test (control: 22 ± 5 versus OA: 13 ± 5 rep.), arm curl test (control: 22 ± 6 versus OA: 18 ± 4), sit and reach test (control: 23 ± 4 versus OA: 12 ± 5; cm), TUG (control: 18 ± 6 versus OA: 29 ± 4; sec), 6-minute walk test (control: 635 ± 142 versus OA: 297 ± 143, m), and balance (control: 18 ± 4 versus OA: 11 ± 3; sec).

**Figure 2:**
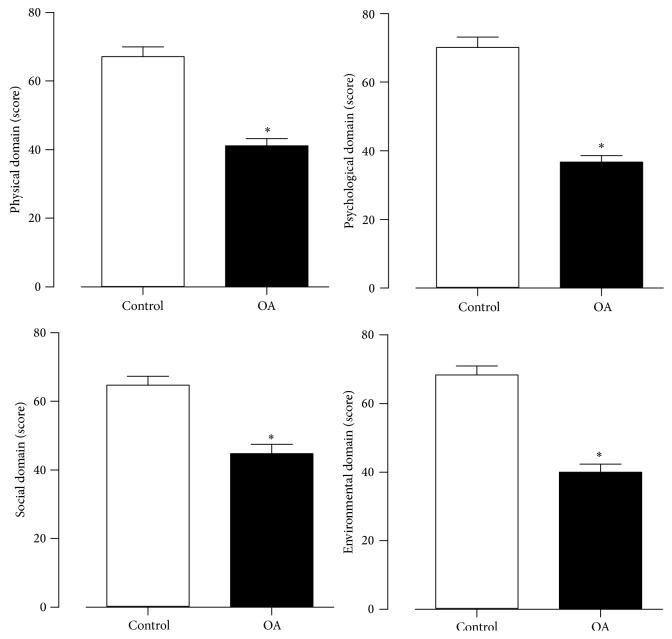
Values expressed in ± SEM of quality of life of control and osteoarthrosis (OA) groups. ^*^ *P* < 0.05.

In all quality of life domains the control (physical: 69 ± 16, psychological: 72 ± 17, environmental: 67 ± 15, and social: 70 ± 15) was higher (*P* < 0.001) than OA (physical: 48 ± 7, psychological: 43 ± 8, environmental: 53 ± 13, and social: 47 ± 14) as shown in Figure 2.

## 4. Discussion

The main objectives of this study were to identify differences in time spent in physical activity, functional fitness, and quality of life with increase in sitting time in older people diagnosed with knee OA. Previous studies [3, 6] suggested that obesity may be considered a risk factor for the development of OA. Falsarella et al. [6] demonstrated that individuals with a high BMI (above 25 kg/m^2^) had an association with joint symptoms; however, Niu et al. [20] demonstrated that obesity is not related to the progression of knee OA.

The decrease in muscle strength is associated with reduced joint flexibility and impairment of functionality, limiting the realization of occupational activity and compromising the welfare on aging [21]. The strength deficits in OA people, evaluated by electrical stimulation of quadriceps muscle by maximal strength voluntary contraction, varied between 15% and 18% on early disease, 24% in level II, according to Kellgren and Lawrence [16], and 38% in level IV [22]. In our study, we found a deficit of muscular strength of 40% in the lower limb when compared to control group. Additionally, our lower limb values (22 ± 5 repetitions) correspond to the findings of previous studies [17, 23].

However, cause-effect relationships between muscle weakness and OA are complex and have been widely debated [10]. Although the muscular strength probably decreases in people with OA as a secondary result of reduced activity, there is evidence that muscle weakness directly contributes to development and progression of OA [24]. Therefore, it may be possible that muscular weakness of the quadriceps could be responsible for functional impairment, predisposing structural damage to the knee, since this muscle acts as a shock absorber in this joint [25].

Ueno et al. [26] reported that changes in older people's flexibility may compromise functional fitness, reducing some activities of daily living. In this study the greater decline in flexibility in OA group was observed. Additionally, Oliveira et al. [2] suggested that improvement in muscular stretching may reduce stiffness and joint pain and improve physical function in osteoarthritis subjects.

The TUG is the most favorable test developed to assess parameters of physical mobility, translated agility, speed, and dynamic balance [18]. Evidence [11] indicates that the knee receptors have an important role in the control of posture, balance, and locomotion capacity. The changes in these receptors may be responsible for the dysfunction on gait pattern in aging, which may be related to muscle reflexes protectors [27]. Although no neural measurements had been done, we considered that alterations may be associated with performance testing changes in OA group.

In relation to aerobic capacity, Jackson et al. [28] showed a reduction by 1% per year and about 50% of this decline is related to inactive lifestyle and a poor body composition. In OA patients, weight loss can lead to significant improvements in reduction of disability and knee burden [5]. Additionally, aerobic exercises can improve VO_2_ peak in this population [5]; however, in the OA group, the time spent in physical activity was lower and sitting time was higher than WHO recommendations. This probably contributed to the poor aerobic fitness levels observed. Similar to our study, Tamegushi et al. [13] demonstrated significant impairment on 6MWT outcome in OA knee patients.

Studies [5, 6, 29] have demonstrated that functional fitness reductions affect significantly self-reported quality of life in the OA older patients. Falsarella et al. [6] indicated that a reduction in joint pain is the most important fact related to this statement; conversely, Alves and Bassitt [29] observed that OA patients have a good quality of life, regardless of functional fitness impairment.

This is a relatively small sample and case control study, with no information about long-term outcomes. Nevertheless, for future public health strategies, this work reinforces the message of the importance of the frequency and regularity of clinical investigation. In addition, there may be some small inaccuracies in estimating maximum aerobic fitness (indirect test). Nevertheless this drawback was common to both the control and OA groups and for all evaluations, thus reducing inaccuracy.

## 5. Conclusion

The present study suggests that knee osteoarthrosis in older women can promote a decline in time spent performing physical activity, functional fitness, and quality of life with consequent increase in sitting time. Thus, despite inherent limitations, our data reinforce the safety and utility of clinical approach strategies that should be considered to address issues observed in this population.

## Figures and Tables

**Table 1:** The radiographic parameters for knee OA diagnosis.

Grade	Criteria

0	Normal
1	Doubtful joint space narrowing, possibly developing osteophytes
2	Definite osteophytes, narrowing missing or questionable joint space
3	Osteophytes moderate, definite narrowing some sclerosis possible joint deformity
4	Large osteophytes marked narrowing sclerosis severe joint deformity established

**Table 2:** Anthropometric parameters.

Parameters	Control	OA	95% of IC	Significance
Age (years)	66 ± 7	67 ± 9	−1.932–4.922	*P* > 0.05
Body mass (kg)	66 ± 7	67 ± 9	−0.631–7.672	*P* > 0.05
Height (cm)	167 ± 0.12	164 ± 0.13	−0.090–0.011	*P* > 0.05
BMI (kg/m^2^)	30 ± 5	33 ± 5	0.690–4.920	*P* < 0.01

Values expressed in mean ± SEM of control and osteoarthrosis (OA) groups. BMI: body mass index.
